# Mechanisms of flagellum construction and maintenance

**DOI:** 10.1186/2046-2530-4-S1-O20

**Published:** 2015-07-13

**Authors:** J Santi-Rocca, T Blisnick, N Ahmed, P Bastin

**Affiliations:** 1 Trypanosome Cell Biology Unit, Institut Pasteur, Paris, France

## 

Cilia and flagella are cylindrical organelles that protrude at the surface of numerous eukaryotes including most human cells. They are composed, from base to tip, of the basal body (9 triplet microtubules), the transition zone (TZ, 9 doublet microtubules) and the axoneme (9 doublet microtubules ± 2 central microtubules). New flagellar sub-units are added at the distal tip by intraflagellar transport (IFT), a dynamic process where IFT motors drag IFT particles in both anterograde and retrograde directions. IFTs concentrate in a pool at the base of the flagellum, whose localization depends on the organism. In Trypanosomatidae, immunofluorescence assays reveal the association of this pool to the TZ, split apart from the cytoplasm by the transitional fibres. In Trypanosoma brucei, we undertook to investigate about the role of the TZ in flagellum formation and maintenance of IFT in mature flagella, by deciphering the role of RP2, a candidate protein located at the TFs [Stephan et al., Traffic 2007]. We constructed a strain impaired in RP2 production by tetracycline-inducible RNAi and obtained the expected phenotype: parasite growth was affected from the third day of induction. In parallel, diminution of IFT concentration at the flagellum base suggests that RP2 is involved in IFT recruitment during flagellum formation and/or RP2 takes part in a ciliary gate that hinders IFT leaking towards the cytoplasm. We are currently studying these hypotheses by live microscopy using fluorescent fusions of IFT proteins, RP2, and other ciliary gate candidates, dissecting the molecular structure and mechanisms involved in flagellum construction and homeostasis.

